# The Influence of Coffee on Reducing Metabolic Dysfunction-Associated Steatotic Liver Disease in Patients With Type 2 Diabetes: A Review

**DOI:** 10.7759/cureus.50118

**Published:** 2023-12-07

**Authors:** Manpreet Kaur, Shanmathi Murugesan, Sachpreet Singh, Katherine N Uy, Jasjeet Kaur, Navina Mann, Rajneet K Sekhon

**Affiliations:** 1 Medicine, University of California, Davis, Davis, USA; 2 Information Technology, Florin High School, Sacramento, USA; 3 Geriatrics, Rajneet K Sekhon MD Inc., Folsom, USA

**Keywords:** type 2 diabetes, hepatic autophagy, chlorogenic acid, obesity and overweight, caffeine intake, fatty liver disease

## Abstract

Metabolic dysfunction-associated steatotic liver disease (MASLD) is a liver disease characterized by hepatic fat accumulation associated with various severities of inflammation and scarring. As studies explore specialized treatments, emerging evidence suggests a potential protective effect of coffee consumption. Consumption of coffee or its components, such as caffeine and/or chlorogenic acid (CA), can reduce markers of liver injury and induce a myriad of other health benefits. However, there is limited research on patients with both MASLD and type 2 diabetes (T2D). Current research suggests that patients with MASLD are at greater risk of developing T2D and future liver-related complications and vice versa. Given that both MASLD and T2D are global burdens, the present literature review analyzes current research to identify trends and determine if coffee can be a viable treatment for MASLD patients with T2D. Results indicate that coffee consumption may protect against MASLD in T2D patients who are overweight/obese through a declined rate of weight gain, inhibition of the mammalian target of rapamycin (*mTOR*) gene, and insignificant changes to the gut microbiome. More longitudinal research on human subjects is needed to establish a causal relationship between coffee consumption and MASLD alleviation.

## Introduction and background

Metabolic dysfunction-associated steatotic liver disease (MASLD) is the most common chronic liver disease globally. A recent meta-analysis reports a steady increase in overall prevalence from 34.8% in 2015 to 38.9% in 2020; the projected prevalence of MASLD in 2040 is reported to be 55.7% [1]. MASLD is characterized by many metabolic factors, including obesity, type 2 diabetes (T2D), triglycerides, and excess fat in the liver not due to alcohol consumption. MASLD ranges from simple steatosis to more severe forms: metabolic dysfunction-associated steatohepatitis (MASH), fibrosis or lobular inflammation, and the most severe stage of cirrhosis defined by permanent shrinking and advanced liver scarring. The permanent liver damage from cirrhosis can progress to liver failure and liver cancer (hepatocellular carcinoma). Liver transplantation is often the available treatment, but multiple studies have explored the recurrence of MASLD following liver transplantation [2,3].

Lifestyle modifications and dietary intervention are the best-known treatment methods to improve hepatic steatosis in patients with MASLD [4]. Other than avoiding or limiting alcohol consumption, patients with MASLD are encouraged to eat at regular time intervals throughout the day, lose weight, limit fructose consumption, and reduce consumption of processed foods [5,6].

As studies explore treatment and preventative modalities, emerging evidence suggests a possible association between coffee and MASLD. Epidemiological questionnaire-based studies conducted between 1993 and 2002 suggest an inverse relationship between high coffee consumption and low levels of γ-glutamyl transferase (GGT) [7]. Population-based studies conducted in Italy within that time frame also show similar results but with more specific markers of liver injury, including alanine aminotransferase (ALT) and alkaline phosphatase (ALP). This exploration comes after evidence of coffee’s role in reducing the risk of all causes of mortality, cardiovascular disease, Parkinson’s disease, several specific cancers, gallstones, and symptomatic gallstone disease [8,9].

MASLD and T2D frequently coexist in patients. A meta-analysis of 80 studies from 20 countries conducted in 2019 found that the global prevalence of MASLD in patients with T2D was 55.5% (95% confidence interval (CI): 47.3-63.7) [9]. Conversely, individuals with MASLD have a 2-3 times increased risk of T2D compared to those without evidence of MASLD [10]. Elucidating a causative relationship between MASLD and T2D is clinically relevant as it would aid in the development of therapeutic approaches. A recently published perspective article suggests that T2D originated from MASLD via an increase in gluconeogenesis [11]. Although further research is warranted to refine the proposed causative relationship, it is a possibility that attenuating MASLD can improve insulin resistance and T2D outcomes. Given an established association between coffee and MASLD, the present literature review aims to clarify if coffee consumption can yield protective liver effects in patients with MASLD and T2D.

## Review

Methodology

PubMed and Google Scholar were the primary databases used in the literature search. The literature review was conducted by all authors in March 2023, prior to the official announcement of the new nomenclature for steatotic liver disease. The key terms searched for were caffeine, coffee, NAFLD, T2D, insulin resistance, and autophagy, used in combinations. Of the articles that included the key terms, only original studies (clinical trials, pilot, experimental, cross-sectional, case-control, etc.) published in the English language between 2013 and 2023 were reviewed. Each study was assessed for its utility, experimental methods, and relevance to the review. The studies considered met the primary objective of the literature review by offering insight into coffee’s association with MASLD in T2D patients. There were no study exclusions based on age, race, ethnicity, country of affiliation, or institution.

**Figure 1:**
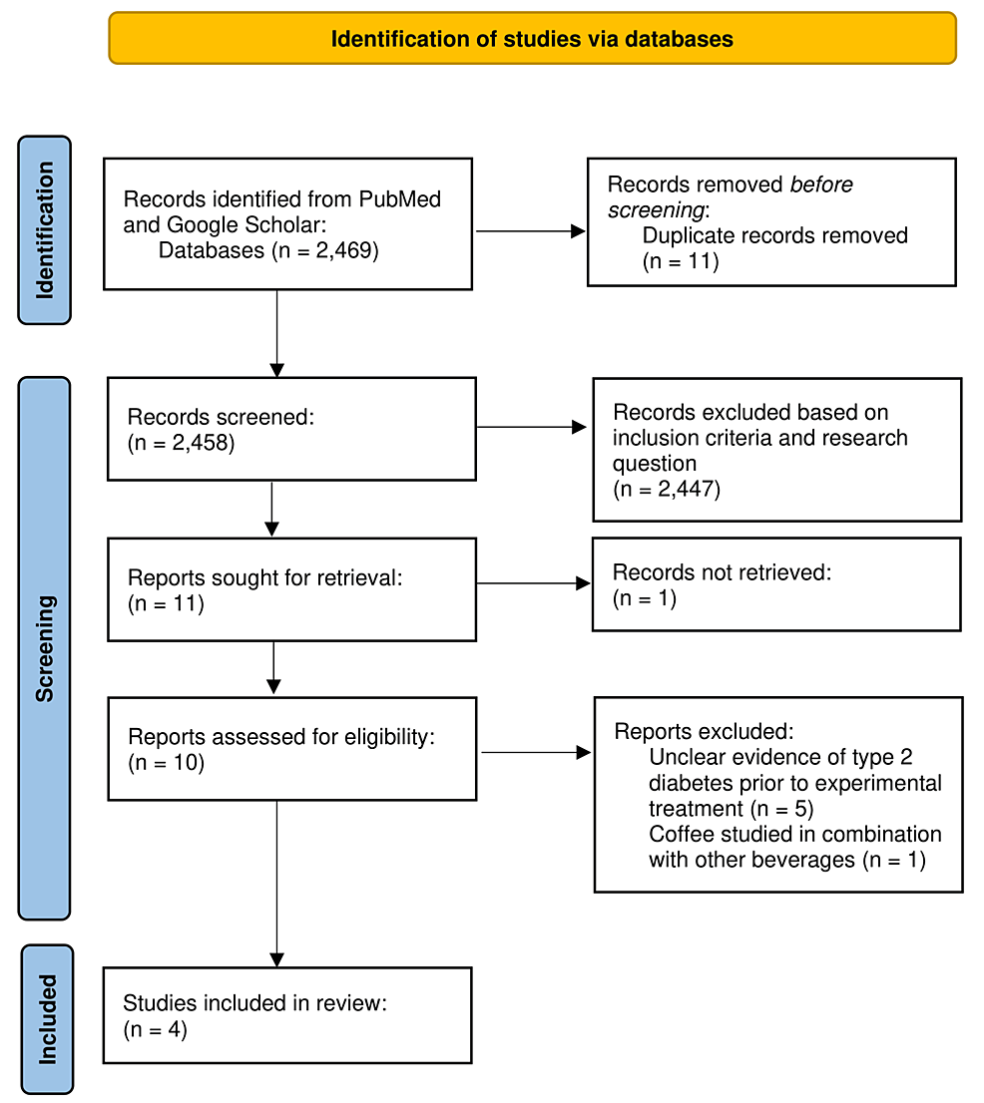
PRISMA flow diagram PRISMA, Preferred Reporting Items for Systematic Reviews and Meta-Analyses

The Preferred Reporting Items for Systematic Reviews and Meta-Analyses (PRISMA) flow diagram is demonstrated in Figure 1.

Diagnosis of MASLD

Noninvasive Markers of MASLD

A diagnosis of MASLD requires evidence of hepatic steatosis on imaging or liver histology exceeding 5%-10% by weight and at least one of the five cardiometabolic risk factors, in the absence of other liver etiologies [12,13]. The five cardiometabolic risk factors include the following: (1) body mass index (BMI) ≥ 25 kg/m^2^ (≥23 kg/m^2^ in Asian) or waist circumference > 94 cm in males and > 80 cm in females, or ethnicity-adjusted equivalent; (2) fasting serum glucose ≥ 100 mg/dL (≥5.6 mmol/L) or two-hour post-load glucose levels ≥ 140 mg/dL (≥7.8 mmol/L) or hemoglobin A1c (HbA1c) ≥ 5.7%(39 mmol/L) or T2D or treatment for T2D; (3) blood pressure ≥ 130/85 mmHg or specific antihypertensive drug treatment; (4) plasma triglycerides ≥ 150 mg/dL (≥1.70 mmol/L) or lipid-lowering treatment; and (5) plasma high-density lipoprotein (HDL) cholesterol < 40 mg/dL (<1 mmol/L) for males and < 50 mg/dL (<1.3 mmol/L) for females or lipid-lowering treatment [13].

MASLD is often asymptomatic, so the diagnosis is usually suspected and affirmed by abnormal serum levels of liver enzymes. While elevated liver enzymes may be temporary, in 2016, the European Association for the Study of the Liver (EASL)-European Association for the Study of Diabetes (EASD)-European Association for the Study of Obesity (EASO) guidelines recommended noninvasive steatosis, MASH, and fibrosis screening for all patients with obesity or diabetes and elevated liver enzymes [14]. In 2020, the American Diabetes Association (ADA) also recommended evaluating T2D patients with elevated liver enzymes or hepatic steatosis for MASH or liver fibrosis [15].

The commonly used noninvasive markers of MASLD are the fatty liver index (FLI), hepatic steatosis index (HSI), and fibrosis-4 (FIB-4) index. The FLI considers blood markers including serum triglycerides and GGT, body mass index (BMI), and waist circumference [16]. In the general population, FLI values ≥ 60 can positively detect hepatic steatosis with a specificity of 86%. The HSI is also proven to accurately predict steatosis since HSI values > 36 can positively detect MASLD with a specificity of 92.4% [17]. However, T2D is a component of the HSI. While the HSI is effective in the general population, it may have less accuracy when screening T2D patients for MASLD. In 2020, the US members of the Global Nonalcoholic Steatohepatitis (NASH) Council recommended a hepatology evaluation for T2D patients with a FIB-4 index score ≥ 1.3 [18]. In a cohort study with 642 T2D patients, fibrosis by imaging was observed in 99 (32.5%) of the 305 T2D patients with FIB-4 index values ≥ 1.3 [19].

Imaging Assessments of MASLD Stages

Noninvasive markers are tools physicians may use to make referrals to a liver specialist. It is recommended to perform imaging or noninvasive procedures to determine the staging or severity of MASLD. Fibroscan, a noninvasive imaging assessment of liver stiffness, is commonly used by primary care physicians and liver specialists to detect the stage of liver fibrosis. Multiple studies have found the Fibroscan to be an accurate tool for assessing liver fibrosis or severe forms of MASLD in T2D patients [20,21].

The liver biopsy remains the gold standard for determining MASH or cirrhosis [22]. Although the procedure has limitations and cannot be performed on all MASLD patients [23], liver biopsy has been proven useful in assessing fibrosis progression in T2D patients compared to the general population with biopsy-proven MASLD [24]. The procedure is suitable when a diagnosis is urgent and the patient is at high risk of disease progression. Noninvasive tools, such as the FLI, FIB-4 index, Fibroscan, or ultrasound imaging detecting hepatic steatosis, should be utilized for initial MASLD assessment.

Biochemical effects of coffee and its main components

**Figure 2:**
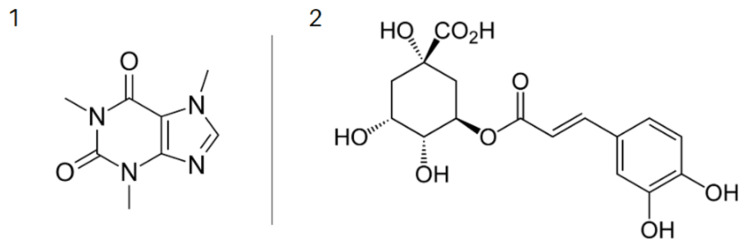
2D chemical structures of caffeine (1) and chlorogenic acid (2)

Coffee contains over 1,000 bioactive compounds, a large number of which contribute to its beneficial effects and strong, bitter taste. The most prominent and widely studied components are caffeine and chlorogenic acid (CA) for their antioxidative properties (Figure 2).

Caffeine is one of the most consumed drugs globally. It accounts for 1%-2% of coffee beans and is present in black and green tea, various chocolate bars, some over-the-counter medications, carbonated soft drinks, and energy drinks [25]. Caffeine reaches 99% absorption in the bloodstream, stomach, and small intestine within 45 minutes, as it is demethylated in the liver into three metabolites: paraxanthine (84%), theobromine (12%), and theophylline (4%) [26]. These metabolites are then excreted in the urine and used quantitatively in several clinical investigations [27].

CA is an active dietary phenolic compound produced from certain plant species during aerobic respiration. Coffee beans are 5%-10% CA by dried weight, a greater percentage than caffeine [25]. CA is also present in apples [28], potatoes [29], and tea [30], among other widely consumed foods in the human diet. In contrast to caffeine, CA is not well absorbed in the digestive tract or small intestine. Only one-third of ingested CA is absorbed from the small intestine into the bloodstream, with the other two-thirds experiencing further metabolism in the colon into compounds, such as caffeic acid and ferulic acid [31-33].

The growing interest in these compounds is attributed to antioxidative properties and anti-inflammatory effects [34], an action that is still not well understood.

Interplay of coffee, MASLD, and T2D

Among studies investigating the role of coffee in alleviating hepatic steatosis or fibrosis, a recent meta-analysis and systematic review of two case-control and four cross-sectional studies revealed an association between reduced hepatic fibrosis (n = 1,074) and regular composition in the MASLD patient population (P = 0.0002) [35]. The meta-analysis included 1,074 patients with hepatic fibrosis and 18,990 patients with hepatic steatosis. This result is consistent with previously mentioned epidemiological prospective studies and case-control studies investigating a reduction or lower risk of MASLD due to coffee consumption.

Studying T2D as well, Coelho et al. [36] conducted a survey-based cross-sectional study to investigate the role of coffee components in the MASLD progression of 98 subjects with T2D (Table 1). All subjects completed a questionnaire on their consumption of caffeinated beverages and foods and provided a 24-hour urine sample for investigation of coffee metabolites. After urine sample collection, all subjects underwent a blood collection and Fibroscan. Pearson’s correlation test showed a negative correlation between total non-caffeine metabolites and the FLI in 86 overweight/obese T2D subjects (P = 0.024). Multiple regression analysis revealed a negative correlation between FLI and total caffeine metabolites (coefficient = -2.507, F = 0.049) and total non-caffeine metabolites (coefficient = -7.057, F = 0.005) in overweight/obese subjects. Coffee consumption was not correlated with MASLD parameters, including the FLI and fibrosis or steatosis severity as indicated by the Fibroscan. Nonetheless, more caffeine metabolites were observed in subjects with no fibrosis compared to fibrotic subjects, indicating that the liver metabolizes more caffeine in the absence of liver inflammation (P ≤ 0.05). The researchers concluded that caffeine may be linked to reduced fibrosis or steatosis in overweight/obese patients with MASLD and T2D.

**Table 1:** Studies on coffee in MASLD and T2D patients MASLD, metabolic dysfunction-associated steatotic liver disease; T2D, type 2 diabetes; BMI, body mass index; LFTs, liver function tests; FLI, fatty liver index; LSM, liver stiffness measurement; CA, chlorogenic acid; CI, confidence interval; CAP, controlled attenuation parameter; CK-18, cytokeratin 18; AST, aspartate aminotransferase; ALT, alanine transaminase; GGT, γ-glutamyl transferase; mTOR, mammalian target of rapamycin; N/A: not applicable/available

Author	Year	Research method	Research subjects	Sample size	Study duration	Intervention	Metric to assess liver function	Results
Coelho et al. [36]	2022	Cross-sectional study	MASLD and T2D patients	98, 86 of whom were overweight/obese (BMI ≥ 25 kg/m^2^)	Retrospective	N/A	Fibroscan, LFTs	The number of total caffeine metabolites per fat-free mass was significantly higher in non-fibrotic patients as compared with fibrotic patients. Multiple regression analysis revealed a negative correlation between FLI and total caffeine metabolites (coefficient = -2.507, F = 0.049) and total non-caffeine metabolites (coefficient = -7.057, F = 0.005) in overweight/obese subjects.
Mansour et al. [37]	2021	Double-blind, placebo-controlled clinical trial	MASLD and T2D patients	100	6 months	Equivalent to 2 cups of coffee/day	Fibroscan, LFTs	Increase in LSM was observed in the supplementation of caffeine plus CA (mean differences: 0.43 KPa (95% CI: -0.46-1.32); P = 0.30), caffeine (mean differences: 0.24 KPa (95% CI: -0.62-1.12); P = 0.57), and CA (mean differences: 0.66 KPa (95% CI: -0.21-1.53); P = 0.09) over the placebo. Treatment by caffeine decreased CAP score (mean differences: -7.45 dB/m (95% CI: -26.48-11.57); P = 0.31), CK-18 fragments (mean differences: -0.13 U/L (95% CI: -0.33-0.08); P = 0.17), AST (mean differences: -0.43 U/L (95% CI: -5.19-4.34); P = 0.87), and ALT (mean differences: -0.12 U/L (95% CI: -4.4-4.13); P = 0.79). Supplementation by caffeine over placebo saw the greatest difference in GGT levels (mean differences: 8.55 U/L (95% CI: -7.05-24.16); P = 0.35) at the end of the study compared to the other groups.
Shokouh et al. [38]	2019	Placebo-controlled experimental study	Zucker diabetic fatty rats	33	10 weeks	Equivalent to 10 cups of coffee/day	Liver triglycerides from liver tissue	After four weeks of intervention, the rate of weight gain lowered in the arabica and robusta groups compared to the control (P = 0.003 and P < 0.001, respectively). Rats treated with arabica (0.56 mmol/L (95% CI: 0.47-0.64); P < 0.001) or robusta (0.62 mmol/L (95% CI: 0.53-0.71); P < 0.001) coffee had significantly lower levels of liver triglycerides at endpoint than the control group (0.79 (95% CI: 0.68-0.90)). Mice in the robusta group had reduced mTOR expression.
Mansour et al. [39]	2020	Pilot randomized placebo-controlled clinical trial	MASLD and T2D patients	26	12 weeks	Equivalent to 2 cups of coffee/day	LFTs	At endpoint, weight reduced significantly in the caffeine plus CA (mean differences: -3.69 (95% CI: -5.18--2.18)), caffeine (mean differences: -0.70 (95% CI: -2.35-0.95)), and CA (mean differences: -0.43 (95% CI: -2.21-1.34)) groups compared to placebo (P = 0.004). Similarly, supplementation with caffeine and/or CA reduced BMI as compared with placebo (P = 0.008). AST and GGT levels declined in the caffeine plus CA, caffeine, and placebo only group. ALT levels only declined in the caffeine plus CA and CA groups. Although statistically insignificant, the number of bifidobacteria increased in the caffeine plus CA treatment group.

A randomized, double-blind, placebo-controlled clinical trial with MASLD and T2D patients published one year prior in 2021 found no evidence of protective effects. Mansour et al. [37] published results showing no reduction of hepatic fat or liver stiffness after CA and/or caffeine intervention. The 100 patients included in data analysis were divided into four treatment groups: (1) 200 mg caffeine and 200 mg CA (CFCA) (n = 27), (2) 200 mg caffeine and 200 mg placebo (starch) (CFPL) (n = 25), (3) 200 mg CA and 200 mg placebo (CAPL) (n = 25), and (4) 200 mg placebo (starch) and 200 mg placebo (PLPL) (n = 23). After six months of intervention, no significant differences in liver stiffness measurements, controlled attenuation parameter (CAP) indicative of fat accumulation, or hepatic blood biomarkers were observed between the four groups. A significant decrease in fasting insulin was observed in subjects receiving CA and caffeine over the placebo (P = 0.01), likely due to discontinued insulin use since participants taking insulin anytime during the six months before treatment were excluded. Overall, this study does not recommend caffeine or CA to treat MASLD in patients with T2D.

Given that different types of coffee contain varying quantities of key components and exhibit unique characteristics, studies have explored the possibility of a particular species showing protective effects. Shokouh et al. [38] studied the effects of robusta and arabica coffee on Zucker diabetic fatty rats with both MASLD and T2D. Thirty-three rats were equally divided into three treatment groups to receive robusta, arabica, or a control for 10 weeks. Within the study, blood samples were collected every third week, and 50 mg of liver tissue was extracted at study completion to assess liver function. Analysis of the liver tissue revealed lower liver triglyceride levels in the robusta (0.62 mmol/L) and arabica (0.56 mmol/L) treatment groups compared to the control group (0.79 mmol/L), indicative of less hepatic steatosis (P < 0.01). After four weeks of treatment, the rate of weight gain in the robusta and arabica groups declined significantly than that of the control group (P = 0.003 and P < 0.001, respectively). Mice in the robusta group had reduced expression of the mechanistic target of rapamycin (mTOR), indicating that the robusta coffee’s unique phytochemical characteristics may play a significant role in gene expression. Although the BMI and specific weights of the mice are unclear, body weight may also be an essential factor mediating coffee-associated effects on the liver. Later findings by Coelho et al. [36] also show that the effect of coffee on the liver may be related to an individual’s body mass.

Prior to publishing the results of the clinical trial, Mansour et al. [39] also studied the effects of caffeine and/or CA on the gut microbiota and metabolic characteristics of MASLD and T2D patients. Twenty-six patients were divided into four intervention groups similar to those of the clinical trial: (1) CFCA (n = 7), (2) CFPL (n = 7), (3) CAPL (n = 6), and (4) PLPL (n = 6). Stool, fasting blood, and three-day food records were collected at the beginning and end of the 12-week study. Results revealed a significant weight (P = 0.004) and BMI (P = 0.008) reduction in patients who took caffeine and/or CA compared to the placebo group. The greatest weight and BMI reductions from baseline levels were observed in the CFCA group, as weight reduced by 3.69 kg (95% CI: -5.18--2.18) and BMI decreased by 1.21 kg/m^2^ (95% CI: -1.67-0.75) post-intervention. Analysis of the gut microbiota revealed an insignificant increase of bifidobacteria in the CFCA group. Bifidobacteria is a bacterial species responsible for fiber digestion and is known to regulate lipid metabolism and reduce intestinal permeability, which could alleviate MASLD [40]. The results indicate caffeine’s role in potentially altering the pathogenesis of MASLD through weight loss and insignificant changes to the gut microbiome.

Results from overweight or obese subjects of MASLD and T2D indicate that coffee may have a protective effect against MASLD. Evidence from the studies by Mansour et al. [37], Shokouh et al. [38], and Mansour et al. [39] suggests that this effect could be modulated by a decline in weight gain, reduced *mTOR* gene expression, or insignificant changes in gut microbiota.

Coffee and increased autophagy in hepatic cells

Autophagy, Insulin Resistance, and MASLD

Autophagy is a process by which a cell selectively recycles damaged cytoplasmic organelles such as mitochondria or unfolded proteins. There are three pathways through which these organelles can be recycled: macroautophagy, microautophagy, and chaperone-mediated autophagy [41]. Environmental stressors such as nutrient deprivation or hypoxia, which leads to oxidative stress, can induce autophagy [42,43]. In macroautophagy, by sensing these environmental stressors, autophagy-related genes form a double membrane structure known as autophagosomes, which engulf the cytoplasmic elements needed to be recycled. Autophagosomes later fuse with lysosomes that contain digestive enzymes and aid in degrading the contents contained in the autophagosomes [41,44]. In microautophagy, the lysosome itself extends toward a damaged organelle and directly digests it [41]. In chaperone-mediated autophagy, selected aggregate proteins are transported into the lysosome with the help of HSC70 (a chaperone protein) and LAMP2A (a lysosomal protein). The impairment of autophagy is associated with multiple dysfunctions in the body, including cancer, cardiovascular diseases, neurodegenerative disorders, and metabolic disorders including diabetes and MASLD [45,46]. In particular, excessive autophagy due to chronic stress can trigger apoptosis [47]. The level of hepatic cell death due to apoptosis undergoes a notable rise in MASH and is aligned with the extent of the severity of the disease [48]. As such, exploring the molecular mechanism of autophagy and how it can be regulated can give valuable insight into potential pharmacological and dietary interventions in preventing the progression of diseases such as MASLD.

One of the key regulators of autophagy is the mTOR complex. In its active state, mTOR suppresses catabolic processes including autophagy by phosphorylating and thus inhibiting Unc-51-like autophagy activating kinase 1 (ULK1) [49-51]. Correspondingly, when mTOR is in its repressed state, ULK1 can induce autophagy [49]. One of the most important signaling pathways that activate mTOR and thus repress autophagy are insulin and insulin-like growth factor signaling pathways [49,52-54]. Higher levels of insulin correspond with the activation of mTOR [49]. In addition, lower blood glucose levels correspond to the inhibition of mTOR [55]. Empagliflozin, a medication widely prescribed to T2D patients, reduces blood glucose concentration by increasing the urinary excretion of glucose [56]. Low glucose levels inhibit mTOR and thus induce autophagy, which reduces endoplasmic reticulum stress and hepatocyte apoptosis, potentially alleviating the progression of MASLD [55].

Caffeine Inhibits mTOR

Caffeine has been shown to inhibit mTOR in induced MASLD models. In an experimental study by Sinha et al. [57] published in 2013, male mice (C57Bl/6) were used in an experiment to study the effects of caffeine on fat metabolism. The mice were either fed a normal chow diet or a high-fat diet (HFD) for four weeks. For the first group, caffeine was injected intraperitoneally daily for three days along with chloroquine (CQ), an autophagy inhibitor, and various analyses were conducted on collected tissues. Additionally, in HFD-fed mice, half were given caffeine in their drinking water for the next four weeks, and their body weight and fat mass were monitored. Tissue samples were analyzed using techniques such as western blotting, polymerase chain reaction, lipidomics, electron microscopy, and histology to assess caffeine’s impact on fat oxidation and autophagy inhibition. Sinha et al. [57] found that all mice who had been given caffeine had reduced levels of mTOR expression and an increase in autophagy compared to mice fed with HFD only. The caffeine-exposed HFD mice, including those only on an HFD, showed hepatic fat loss and reduced levels of intrahepatic lipids. In order to achieve similar results in humans, Sinha et al. [57] propose that 2-3 cups of coffee would need to be consumed per day.

Shokouh et al. [38] observed a similar effect on Zucker diabetic fatty rats with both MASLD and T2D. The consumption of coffee from the robusta species, a species with the highest level of caffeine among all known species, resulted in a onefold downregulation of mTOR compared to rats who had been given arabica coffee. Overactivation of mTOR can inhibit insulin receptor substrate 1, which leads to insulin resistance in hepatocytes. Thus, by lowering levels of active mTOR, caffeine could potentially improve insulin sensitivity. The molecular mechanism through which caffeine downregulates mTOR in MASLD and T2D is yet to be explored. While preclinical studies provide promising insights, further research with human subjects is needed to explore this phenomenon.

## Conclusions

The present literature review finds that there may be a potential benefit to coffee consumption for overweight/obese patients of MASLD and T2D. The protective effect against MASLD may be mechanistically related to a reduced rate of weight gain, a current treatment option for MASLD, mTOR inhibition, and an altered gut microbiome. While there is conflict if all patients with MASLD and T2D can benefit from coffee consumption, current studies offer a strong foundation. Further longitudinal research with human subjects that includes weight measurements, histological samples, and gut microbiome samples is needed before clinical recommendations can be made. The potential for caffeine to be a lifestyle modification for overweight/obese MASLD and T2D patients due to its modulation of mTOR expression and autophagy-inducing effects should also be investigated in future research.
